# Hepatic Retinyl Ester Hydrolases and the Mobilization of Retinyl Ester Stores

**DOI:** 10.3390/nu9010013

**Published:** 2016-12-27

**Authors:** Lukas Grumet, Ulrike Taschler, Achim Lass

**Affiliations:** Institute of Molecular Biosciences, University of Graz, Heinrichstraße 31, 8010 Graz, Austria; lgrumet@gmail.com (L.G.); ulrike.taschler@uni-graz.at (U.T.)

**Keywords:** retinyl ester hydrolase, liver, hepatocyte, hepatic stellate cells, lipid droplet, mobilization

## Abstract

For mammals, vitamin A (retinol and metabolites) is an essential micronutrient that is required for the maintenance of life. Mammals cannot synthesize vitamin A but have to obtain it from their diet. Resorbed dietary vitamin A is stored in large quantities in the form of retinyl esters (REs) in cytosolic lipid droplets of cells to ensure a constant supply of the body. The largest quantities of REs are stored in the liver, comprising around 80% of the body’s total vitamin A content. These hepatic vitamin A stores are known to be mobilized under times of insufficient dietary vitamin A intake but also under pathological conditions such as chronic alcohol consumption and different forms of liver diseases. The mobilization of REs requires the activity of RE hydrolases. It is astounding that despite their physiological significance little is known about their identities as well as about factors or stimuli which lead to their activation and consequently to the mobilization of hepatic RE stores. In this review, we focus on the recent advances for the understanding of hepatic RE hydrolases and discuss pathological conditions which lead to the mobilization of hepatic RE stores.

## 1. Introduction

The turnover of vitamin A (retinol and metabolites) involves two major metabolites, retinol and the esterified form of retinol, retinyl ester (RE). These two forms are interchangeable by enzymatic reactions: REs are hydrolyzed to retinol and fatty acids by enzymes named RE hydrolases, whereas retinol is esterified to REs by acyltransferases. These hydrolysis and re-esterification reactions occur in several tissues and cell types [1]. One example is the intestinal uptake of dietary REs [2]: prior to their uptake, dietary REs require hydrolyzation to retinol in the lumen of the intestine. Only retinol and not REs is taken up from enterocytes. In enterocytes, retinol is esterified to REs and packed into chylomicrons for secretion. Another example is the hepatic utilization of vitamin A [3]: in liver, hepatocytes take up RE-containing chylomicron remnants via the endocytic pathway. Within endosomes/lysosomes, REs are hydrolyzed and transferred to the endoplasmic reticulum (ER). Retinol is then esterified to REs and stored in cytosolic lipid droplets (LDs) of hepatocytes and even more so in hepatic stellate cells (HSCs). Upon demand, these hepatic RE stores are mobilized and retinol, bound to its specific transport protein retinol-binding protein 4 (RBP4), is released into circulation. Circulating retinol is utilized from peripheral tissues for the generation of the nuclear receptor ligand retinoic acid to exert biological activities through gene regulation events [4]. Circulating retinol is utilized by the retinal epithelium for the generation of a chromophore, 11-*cis*-retinal, required for the visual cycle thereby enabling vision [5]. Excessive retinol is stored as REs mainly in the liver and to a lesser extent in other tissues such as adipose tissue, lung, and intestine [6].

In the liver, the largest quantities of REs are stored in cytosolic LDs of a specialized cell type, the HSCs [7]. The mobilization of hepatic RE stores requires the activity of RE hydrolases. Hepatic RE stores are essential for maintaining constant plasma retinol levels (2–3 µM in humans, 1–1.5 µM in rodents [8]). Furthermore, hepatic RE stores are also mobilized under times of insufficient vitamin A intake [9,10], and upon certain types of liver diseases [11,12,13]. Despite this essential role of hepatic RE hydrolases, the identity of enzymes responsible for the hydrolysis of RE stores is largely unknown. In the next sections, we provide a brief overview on the role of different liver cell types in hepatic vitamin A turnover and summarize the recent advances in the knowledge on hepatic RE hydrolases. Furthermore, we discuss exemplified pathological conditions which lead to the mobilization of hepatic RE stores.

## 2. Brief Overview of the Role of Different Liver Cell Types in Hepatic Vitamin A Turnover

The liver consists of several cell types which are known to contribute to vitamin A turnover [14]. In general, the different liver cell types can be divided into two main groups, the parenchymal and non-parenchymal cells [15]. The vast majority of liver cells are parenchymal cells, also termed hepatocytes, which account for ~78% of the liver volume [16]. These cells are known to perform most of the liver’s functions in carbohydrate, fat, bile acid, and protein metabolism [15].

### 2.1. Parenchymal Cells in Vitamin A Turnover

Hepatocytes do not store much hepatic vitamin A since they only contain ~1.7% hepatic retinoids [17]. Despite this low retinoid content, they play an important role in hepatic vitamin A turnover: hepatocytes take up dietary vitamin A, contained in chylomicron remnants, from the circulation [18]. In fact, hepatocytes take up the majority of ^3^H-labeled chylomicron remnants (around 65%) within 30 min of injection [19]. Chylomicrons derive from the intestinal fat absorption, where vitamin A is packed into chylomicron particles and released via the lymph into the circulation [20]. Circulating chylomicrons are depleted from triglycerides and to a lesser extent from REs (~80% and ~25% of initial triglyceride and RE content, respectively) by the action of lipoprotein lipase (LPL) and hepatic lipase [21]. Only chylomicron remnants can pass through the fenestrated sinusoidal endothelial cell lining to enter the space of Disse [22]. (For a detailed description of nutritional retinoid uptake and delivery to the liver see [7,14].) In addition to the uptake of chylomicron remnants through the endocytic pathway, hepatocytes also take up unesterified/free retinol, transported by its specific binding protein RBP4, termed as holo-RBP4. In fact, hepatocytes exhibit specific binding sites for RBP4 [23,24]. Two RBP4 receptors are known to facilitate the transport of retinol:RBP4 complexes across cellular membranes [25,26]. However, the expression of the stimulated by retinoic acid 6 (STRA6) protein-coding gene is very low or absent in the liver [27]. The expression of the second receptor, RBP4 receptor-2 (RBPR2), is suppressed by retinol and retinoic acid and inversely correlates with liver vitamin A content [26]. Thus, when intracellular retinol levels are high, RBPR2 is thought not to facilitate retinol uptake into hepatocytes. The significance of the holo-RBP4 uptake was questioned by a study employing a RBP4-deficient mouse model expressing human RBP4 (hRBP4) selectively in muscle, demonstrating that circulating hRBP4 is not taken up by hepatocytes [28]. Since addition of retinol is known to stimulate RBP4 secretion of hepatocytes [29], the available data suggest that hepatocytes rather secrete than take up retinol under vitamin A-sufficient conditions. (For more detail on hepatic RBP4 metabolism see [7,30].)

Within hepatocytes, chylomicron-contained REs are hydrolyzed to retinol and fatty acids in the endocytic system, supposedly in early and late endosomes, and not lysosomes [31]. In the cytosol, free retinol exists mostly bound to cellular retinol-binding protein [32]. At the ER, these retinol:cellular retinol-binding protein complexes are the substrate for the subsequent esterification reaction, thereby generating REs which are deposited in cytosolic LDs [33]. Conversely, apo-cellular retinol-binding protein (“apo” precedes the binding protein when devoid of the ligand) has been shown to stimulate the hydrolysis of REs of ER membranes [34]. Furthermore, the actual amount of REs stored in hepatocytes is rather limited. For example, rat hepatocytes have been reported to contain around 9% of hepatic REs, while HSCs contain roughly 88% thereof [17]. Interestingly, the fate of retinol which derives from the endocytic system in hepatocytes is known to depend on the vitamin A status. In times of vitamin A surplus, the majority of retinol is transferred from hepatocytes to non-parenchymal stellate cells for storage [11,35]. This transfer of retinol from hepatocytes to HSCs involves the hydrolysis of REs to retinol in hepatocytes, presumably in the endocytic system. REs, which are taken up from chylomicron remnants, are not directly transferred to HSCs [36]. In contrast, in times of vitamin A deficiency, hepatocytes secrete retinol as RBP4:retinol complexes to the circulation for uptake by peripheral tissues [30]. In addition to cytosolic LDs, hepatocytes are also known to contain LDs in the lumen of the ER [37]. These lipid reservoirs are thought to be channeled to very-low density lipoprotein assembly and secretion [38,39]. Thus, REs in hepatocytes may also be stored in such luminal LDs of the ER. Thus far, however, the RE content of luminal LDs of the ER has not been determined.

### 2.2. Non-Parenchymal Stellate Cells in Vitamin A Turnover

In comparison to parenchymal cells, which account for 78% of the liver volume, non-parenchymal liver cells contribute only 6.3% to the liver volume, the rest being the extracellular space compartment [16]. Non-parenchymal cells are mostly composed of sinusoidal endothelial cells, Kupffer cells, and HSCs. Although HSCs account for only 1.4% of the liver volume [16], they are most prominent in vitamin A turnover since they contain the largest quantities of vitamin A [14,17]. HSCs are located in the space of Disse between the non-parenchymal sinusoidal endothelial cells and parenchymal cells. They comprise around 5%–8% of all liver cells [16,35,40]. HSCs have been initially named lipocytes or fat-storing cells because of their high lipid content [35]. LDs of these cells have been shown to contain comparable amounts of REs, cholesteryl esters and triglycerides [41]. These cells contain most of the hepatic REs. In fact, around 70%–95% of all RE contained in the liver are stored in cytosolic LDs of HSCs [41].

HSCs have been shown to take up retinol bound to RBP4 as well as bound to albumin [1,42]. Interestingly, the intracellular fate of retinol in HSCs varies depending on the type of carrier protein. RBP4-delivered retinol localizes to the cytosol, the ER membrane as well as the cytosolic LDs [42]. Albumin-delivered retinol remains primarily associated with the plasma membrane [42]. Furthermore, HSCs also secrete retinol; however, this process is not dependent on the presence of RBP4 [43]. A co-culture of HSCs with hepatocytes increases the release and transfer of retinol from HSCs to hepatocytes [43]. Yet, HSCs also release retinol directly into the culture media suggesting that in vivo HSCs might mobilize retinol directly into the circulation, a process which might not require the transfer to parenchymal cells for secretion [44].

The unique feature of HSCs to undergo activation has attracted much attention. Once quiescent HSCs are activated, they transform into myocyte-like cells and lose their entire RE content [13]. This process of HSC activation is a known hallmark for the progression of liver injury to the onset of fibrosis, and is triggered by a number of mediators including e.g., mitogenic and fibrogenic cytokines [45]. Activated HSCs excrete extracellular matrix proteins such as collagens which contribute to the scar-forming process in which fibrous tissue replaces injured liver tissue [13,46]. To a certain degree, depending on the disease stage, fibrous liver tissue can undergo resolution [47]. The observation that one of the early hallmarks of HSC activation is the loss of their RE stores has nourished the search for the molecular mechanism and lastly for the identification of RE hydrolases as discussed in the following sections.

### 2.3. Other Non-Parenchymal Cell Types

Kupffer and sinusoidal endothelial cells are by number the largest group of non-parenchymal liver cells and account for 2.8% and 2.1% of liver volume, respectively [16]. Kupffer cells are actually liver-residing tissue-macrophages [48] with a high endocytic and phagocytic capacity clearing gut-derived particulate matter and soluble bacterial products [49]. They adhere to the lining of endothelial cells within the sinusoid where they come first in contact with bacterial debris and endotoxins (e.g., lipopolysaccharide).

Kupffer cells have been shown to contain around 1.4% of hepatic retinoids (expressed as per cells) which is comparable to that of parenchymal cells [17]. Earlier studies reported higher vitamin A content in the Kupffer cell fraction (~12% of hepatic vitamin A) [50]. Kupffer cells contain chylomicron remnant recognition sites which are in their properties distinct to those of parenchymal cells [51]. In vitro, Kupffer cells take up more chylomicron remnants than hepatocytes when calculated on a per cell basis [52]. Due to their low number in liver, their contribution to total hepatic chylomicron remnants uptake is obviously very low. Injection of radiolabeled chylomicron remnants into rats yielded an accumulation of 8.6% of radioactivity in Kupffer cells as compared to 80% in parenchymal cells [51]. The distinct nature of chylomicron remnant binding sites of Kupffer cells gave rise to the speculation that Kupffer cells might rather take up a certain subpopulation of chylomicron remnants and thereby function as a protection system against potentially atherogenic chylomicron remnant particles [51]. In addition to their uptake of chylomicron remnants, Kupffer cells have also been shown to take up RBP4. Kupffer cells take up less than 5% of radiolabeled RBP4 of all non-parenchymal cells (determined in situ) indicating that Kupffer cells do not contribute much to the uptake of circulating retinol [23]. Kupffer cells harbor RE hydrolase activity which is ~2.5-fold higher than that of parenchymal cells but ~6-fold lower than that of HSCs (expressed as activity per cell protein) [17]. Kupffer cells contain similar amounts of RBP4 as HSCs, but ~2-fold lower amounts than parenchymal cells (expressed as per mg cell protein). Across the liver cell types, parenchymal cells contain most of the RBP4 and HSCs, as well as most of the REs. In comparison to other liver cell types, RBP4 and RE levels of Kupffer cells are much lower, which may suggest that Kupffer cells do not contribute much to RBP4 secretion and vitamin A storage. This view of a neglectable role of Kupffer cells in hepatic vitamin A metabolism, however, has been recently challenged by the phenotype of lysosomal acid lipase (LAL)-deficient mice [53]. These mice show massive accumulation of neutral lipids (mostly cholesteryl esters) in Kupffer cells [53] suggesting that, at least under pathological conditions, Kupffer cells accumulate large amounts of neutral lipids presumably also including vitamin A.

The third non-parenchymal cells are the sinusoidal endothelial cells. As the name implies, these endothelial cells shape the lining of the hepatic sinusoid [54]. They form small fenestrations (50–300 nm in diameter) between the blood and the hepatocyte surface [22]. These fenestrations act as filters that allow diffusion of substances typically smaller than of the size of chylomicrons (100–1000 nm). In contrast to chylomicrons, chylomicron remnants (90–250 nm) pass through the fenestrations and are readily taken up by liver parenchyma [22]. Furthermore, sinusoidal endothelial cells have also been acknowledged for their endocytic capacity, for their scavenging function, for their role in liver immunity and in the secretion of cytokines, eicosanoids, and extracellular matrix components [54]. Similar to Kupffer cells, also sinusoidal endothelial cells contain low levels of REs which are comparable to that of parenchymal cells [17]. Endothelial cells also harbor RE hydrolase activity which is the lowest of all liver cell types and around 14 times lower (expressed as activity per cell protein) than that of HSCs [17]. Of all liver cell types, sinusoidal endothelial cells contain the least amount of RBP4 [17], suggesting that they may not contribute much to vitamin A secretion in the liver.

**Figure 1:**
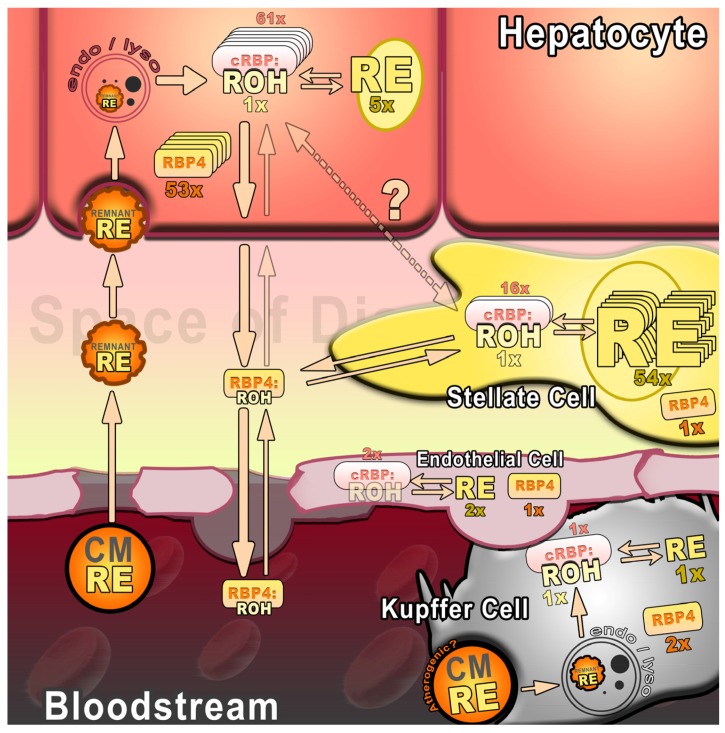
Schematic representation of hepatic vitamin A turnover. Circulating retinyl esters (REs) contained in chylomicron (CM) remnants and retinol:retinol-binding protein 4 complexes (ROH:RBP4) pass endothelial fenestrations and enter the space of Disse. CM remnants are taken up via endocytosis and cleared in endosomes/lysosomes (endo/lyso) by various cell types. Intracellularly, ROH, bound to cellular retinol-binding protein (cRBP:ROH), is esterified to RE or secreted as ROH:RBP4 complexes. Arrows indicate metabolic fluxes and are adapted from [55,56]. Relative amounts of retinoids (ROH + RE) and retinol-binding proteins (cRBP and RBP4) are indicated by fold difference (x = fold) between cell types (on a per cell basis as determined in [57]). To improve clarity, differences larger than 10× are additionally indicated by reduplicated symbols in increments of ten. Note: The relative amount of liver cells (hepatocytes, stellate, Kupffer, and endothelial cells, comprising roughly 78%, 1.4%, 2.8%, and 2.1% of liver volume, respectively [16]), is not reflected in the figure.

Figure 1 depicts a simplified illustration of the interplay of different liver cell types in hepatic vitamin A turnover. Nutritional vitamin A, contained mostly in chylomicron remnants, is endocytosed by hepatocytes. Once cleared in the endo-/lysosome, retinol bound to RBP4 is mostly released to the space of Disse. Retinol:RBP4 complexes may be taken up by HSCs for storage or distributed to peripheral tissues via the blood stream. Also, Kupffer and endothelial cells contribute to vitamin A turnover. Yet, HSCs contain most hepatic REs while hepatocytes exhibit the highest RBP4 content (as indicated by fold differences, x = fold), indicative for a prominent role in RE storage and retinol release, respectively.

## 3. Hepatic Retinyl Ester (RE) Hydrolases

### 3.1. Retinyl Ester Hydrolases of Hepatocytes

#### 3.1.1. Retinyl Ester Hydrolases in the Endocytic System of Hepatocytes

Hepatocytes take up vitamin A either as retinol, bound to RBP4 [1], or as REs, contained in chylomicron remnants [7]. Chylomicron remnants are known to be internalized as whole particles via endocytosis (for detailed review on hepatic chylomicron uptake see [18]). Once in endosomes, chylomicron remnants are further processed, as endosomes mature into late endosomes/lysosomes [58]. It is known that the endocytic system contains both neutral and acid bile salt-independent RE hydrolase activities [59]. However, the proteins responsible for the hydrolysis of REs in this process are not well established. LPL and hepatic lipase have been shown to be internalized together with chylomicron remnants [60], while only LPL is known to exhibit RE hydrolase activity [61]. A third protein, rat serum carboxylesterase ES-2, was proposed to catalyze the hydrolysis of REs after internalization of the remnants in early endosomes [62]. ES-2 exhibits RE hydrolase activity at neutral pH [63], is solely expressed in the liver and secreted by primary rat hepatocytes into serum [64], and lacks the ER retention consensus tetrapeptide sequence histidine-any amino acid-glutamate-leucine (HXEL) [64,65]. Yet, its internalization has so far not been demonstrated. Both proteins are implicated in the extracellular RE hydrolysis of chylomicron remnants in the space of Disse and in early endosomes. Although it appears plausible that LPL and ES-2 are internalized during the uptake of chylomicron remnants, any role in the endosomal hydrolysis of REs has so far not been established.

Secretory bile-salt dependent carboxylester hydrolase/lipase (CEL) is known to hydrolyze REs at neutral pH, requiring mMolar concentrations of bile salts [66,67]. CEL is synthesized in the rat pancreas and liver [66], and human hepatoma cells secrete more enzyme into the medium than they retain [68]. Secreted hepatic CEL may hydrolyze REs contained in chylomicron remnant in the space of Disse. A more recent observation that CEL is internalized into the endosomal system, at least under pathological conditions such as diabetes [69], suggests that this enzyme may—under certain conditions—play a role in the neutral hydrolysis of REs in endosomes. Under normo-physiological conditions, however, CEL apparently does not play a role in the hydrolysis of chylomicron-contained REs since overexpression in rat hepatoma cells as well as deficiency in mice has no impact on hepatic uptake or metabolism of chylomicron-REs [70].

Two proteins have been found to hydrolyze REs at acidic pH and have been suggested to function as RE hydrolases in lysosomes: LAL and carboxylesterase ES-10 [71,72]. LAL is well established to be limiting for the acid hydrolysis of cholesteryl esters and triglycerides [73]. Humans and mice deficient in LAL activity develop a phenotype known as cholesteryl ester storage disease [73,74]. Earlier reports suggested that two different enzymes are responsible for the hydrolysis of REs and cholesteryl esters in lysosomes, since acid cholesteryl ester but not acid RE hydrolase activity of liver lysates was sensitive to mM concentrations of ionic halides (e.g., CaCl_2_) and cholesterol oleate did not inhibit the acid RE hydrolase activity [75]. A recent study, however, has challenged this concept: Grumet et al. [72] showed that incubation of human hepatoma cells HepG2 with RE-enriched lipoproteins and the LAL inhibitor LALISTAT 2 led to increased RE content in lysosomal fractions indicating that LAL is involved in the clearance of endocytosed REs. Surprisingly, the vitamin A phenotype of LAL-deficient mice seemingly contradicts this observation [72]. In fact these mice showed decreased RE concentrations in the liver [72]. This observation, however, may not necessarily indicate that LAL does not play a role in lysosomal clearance of REs in hepatocytes. Histological analyses of livers of LAL-deficient mice showed massive accumulation of cholesteryl esters (even needle-shaped cholesterol crystals deposition) in Kupffer cells rather than hepatocytes [53]. This could be explained by inflammatory processes that led to Kupffer cell activation. As a consequence, Kupffer cells may internalize neutral lipids which presumably originate from hepatocytes. Similar processes which could lead to Kupffer cell activation could also activate HSCs which would lead to their transformation and the loss of their RE store. This could explain decreased hepatic RE concentration of LAL-deficient mice which was accompanied by increased circulating retinol and RBP4 levels [72]. Future studies employing a hepatocyte-specific LAL-deficient mouse model would allow to delineate any role of LAL in vitamin A turnover of hepatocytes.

Carboxylesterase ES-10 is the second enzyme known to exhibit acid RE hydrolase activity [71]. In addition to acid RE hydrolase activity, ES-10 also exhibits neutral RE hydrolase activity. Because of its acid hydrolysis properties, ES-10 is suggested to be involved in the RE breakdown in endosomes/lysosomes and/or the ER [71]. However, any functional role of ES-10 in lysosomal RE hydrolysis has not been established.

#### 3.1.2. Retinyl Ester Hydrolases of the Endoplasmic Reticulum of Hepatocytes

The mechanisms how REs are transferred from the endocytic system to the ER are not well characterized. Subcellular fractionation studies, after injecting ^3^H-labeled RE containing chylomicrons into rats, showed that the radioactivity first appeared within early endosomal fractions and co-migrated time-dependently with endosomal fractions as well as the ER and much less with lysosomal fractions [31,76]. These observations suggested that endocytosed REs are hydrolyzed in early/late endosomes or are directly transferred from the endosomes to the ER, not involving their hydrolysis and bypassing lysosomes. The direct transfer of REs from endosomes to the ER, without their hydrolysis, appears rather unlikely since at least in J774 macrophages, when incubated with ^3^H-labeled chylomicron REs, the radioactivity appeared predominantly in early endosomes and was time-dependently converted to ^3^H-retinol, present in the cytosol [77].

The ER is known to exhibit both RE anabolizing and catabolizing activities. At the ER, lecithin:retinol acyltransferase (LRAT) and diacylglycerol:acyltransferase 1 (DGAT1) are known to catalyze the conversion of retinol to REs [78,79]. Retinol bound to cRBP is the substrate for the LRAT reaction [33]. It is evident from studies in LRAT-deficient mice that LRAT is the major retinol acyltransferase in liver [80,81]. Since its expression in hepatocytes is very low or absent [82], LRAT may rather account for retinol-esterifying activity in HSCs. DGAT1 catalyzes the acyl-CoA:retinol acyltransferase (ARAT) reaction [78]. Since murine DGAT1-ko livers exert markedly reduced ARAT activity which is accompanied by increased retinol levels [78], it suggests that DGAT1 accounts for the majority of hepatic ARAT activity.

For the hydrolysis of REs at the ER of hepatocytes a number of esterases/carboxylesterases (ESs/CESs) have been identified. The murine, rat, and human genome contain at least 20, 15, and 5 protein-coding CES genes, respectively [83]. The murine and rat genome encode for more CES genes which is likely a result of gene duplication events. Together they comprise the mammalian non-specific carboxylesterase super-gene family (EC 3.1.1.1). Because of the high number of genes and splice variants, CESs may comprise a highly redundant protein family. Originally, they have been numbered rather following their discovery, irrespective of the species, while later it turned out that some of them were actually orthologue genes or splice variants. A new nomenclature has been developed for simplification, which follows the nomenclature of the human CES1 to 5 genes, with splice variants numbered as A1 to A3 (e.g., CES1A1 to CESA3). Since rat and murine genomes encode additional genes and in order to differentiate between human and rodent genes the “A” for human genes was replaced by “a” for rodent genes and extend up to “h” [83].

Many of the CES members are highly expressed in the liver, encode an ER retention signal (HXEL) at the C-terminus, and localize to the ER [84]. In vitro RE hydrolase activities have been found for the following members of this gene family: rat ES-2 (annotated as Ces2c) [63], ES-3 (annotated as Ces1e) [85], ES-4 (annotated as Ces1f) [85], ES-10 (annotated as Ces1d) [71], murine ES-22 (annotated as Ces1e, homologous to rat ES-3) [86], and pig ES-4 [87]. From these liver CESs, a role in RE metabolism has been demonstrated only for ES-22 by overexpression studies in cells [86]. ES-22 is highly expressed in hepatocytes and not in HSCs, carries an HXEL sequence at the C-terminus, and localizes to the ER [86]. Its localization to the ER argues for a role in counteracting the esterification of retinol by acyltransferases. Since the availability of retinol was shown to induce RBP4 secretion [29,88], presumably from the ER to the Golgi complex, it can be hypothesized that RE hydrolases at the ER, such as ES-22, will promote retinol/RBP4 secretion.

Among several CESs, ES-10 shows highest expression levels in liver and hepatocytes followed by ES-4 and ES-3 [71,89]. These observations suggest that ES-10 may play a major role in RE hydrolysis in liver. In fact, it was estimated from purification studies of liver microsomal CESs that rat ES-10, ES-4, and ES-3 account for 60%, 34%, and 3% of neutral RE hydrolase activity, respectively [90]. However, the physiological relevance of any of these enzymes is currently unclear and remains to be demonstrated by overexpression and knock-out studies. RE hydrolases at the ER are thought to represent a switch-point between retinol release and retinol esterification which subsequently might be stored in cytosolic LDs. (For a detailed review on the roles of CESs in hepatic RE turnover see [59,62].)

#### 3.1.3. Retinyl Ester Hydrolases of the Lipid Droplet of Hepatocytes

LDs are known to harbor two established lipases which are capable of hydrolyzing REs. The first LD-associated hydrolase, hormone-sensitive lipase (HSL), is a multifunctional enzyme that has been demonstrated to be rate-limiting in the mobilization of REs of the white adipose tissues [91]. In contrast to the adipose tissue the expression level of HSL in liver is very low [92]. A recent study reported that HSL is particularly enriched in hepatocytes and much less expressed in non-parenchymal cells [93]. While the authors demonstrated decreased cholesteryl ester hydrolase activity accompanied by increased cholesteryl ester content in primary hepatocytes derived from HSL-deficient mice [93], no data are available in regard to hepatic RE content. Thus, the role of HSL in liver RE metabolism and in particular in hepatocytes is unknown and remains to be examined.

The second LD-associated hydrolase is adipose triglyceride lipase (ATGL). ATGL has been shown to be expressed in murine hepatocytes and human hepatic carcinoma cells and to localize to LDs in HepG2 cells [94]. Furthermore, ATGL together with its binding protein pigment epithelium-derived factor affects triglyceride catabolism in hepatocytes [94]. Although ATGL has been shown in one study [95] but not another [96] to affect RE catabolism in primary HSCs, any role in RE catabolism of hepatocytes has not been demonstrated.

In addition to the established LD-associated lipases, HSL and ATGL, CES3 and CES31 have been detected on LD preparations of murine primary hepatocytes [97]. CES3 (annotated as Ces1d, also known as triglyceride hydrolase, TGH) has been shown to be expressed in parenchymal cells which surround the capillary vessels leading to the central vein [98], and not Kupffer or endothelial cells [99] in mice. CES3 has an established role in the mobilization of intracellular triglyceride stores for very low density lipoprotein secretion [100,101,102] (for review see [103]), but any RE hydrolase activity for the murine protein has not been reported. Similarly, also for murine CES31 (annotated as Ces3a), any role in RE catabolism is unknown.

The mobilization of REs contained in cytosolic LDs of hepatocytes may, under certain conditions, also involve acid hydrolysis. Entrapment of cytosolic LDs by the phagophore membrane of autophagosomes and fusion with lysosomes delivers the cargo to lysosomes and results in the formation of autolysosomes [104,105], a process termed lipophagy [106]. Under fasting, lipophagy contributes significantly to the breakdown of hepatic triglycerides, contained in cytosolic LDs, since inhibition of autophagy protein 5 (ATG5) leads to triglyceride accumulation in hepatocytes and a phenotype similar to non-alcoholic fatty liver disease (NAFLD) [105,107]. Since LDs of hepatocytes also contain REs, it suggests that also REs might undergo lipophagy. The contribution of lipophagy to overall RE hydrolysis in hepatocytes, however, has not yet been investigated.

### 3.2. RE Hydrolases of Hepatic Stellate Cells

HSCs contain multiple LDs of various sizes which have been classified into type I and type II [41]. Type I LDs are membrane-associated and may derive from multivesicular bodies, and thus may represent lysosomes [35]. Type II LDs are much larger than type I LDs and do not associate with membranes, and thus may represent cytosolic LDs [35].

The breakdown of triglycerides, stored in cytosolic LDs, is termed lipolysis and has been intensively investigated in a tissue type specialized for energy storage, the adipose tissue [108]. Lipolysis is known to involve several enzymes which act consecutively in a lipolytic cascade [109]. From this classical lipolytic cascade (three enzymes for a three-step reaction [109]), only ATGL together with its coactivator protein comparative gene identification-58 (CGI-58), and not HSL or monoglyceride lipase (MGL), have been identified on lipid droplets of HSCs [110]. A role of ATGL in HSC RE mobilization is evident from several observations [95]: (i) ATGL exhibits RE hydrolase activity which is stimulated by the presence of its coactivator protein CGI-58; (ii) Primary HSCs incubated with a pharmacological inhibitor for ATGL as well as primary HSCs from ATGL-deficient mice show increased RE content; (iii) Primary HSCs of ATGL-deficient mice accumulate more REs after overnight retinol loading in comparison to control HSCs. Furthermore, upon serum starvation of these retinol-loaded HSCs, ATGL-deficient HSCs lose less cellular REs than control HSCs. Together, these results indicate that ATGL is involved but not limiting for RE mobilization of HSCs. This view is bolstered by the observation that ATGL-ko mice exhibit unaltered hepatic RE content [95]. A minor role of ATGL in RE mobilization of HSCs was also suggested by Tuohetahuntila et al. [96] since in their study in vitro activation of primary ATGL-deficient HSCs resulted in an attenuated loss of triglycerides but not REs.

Gene expression analyses of 12 CESs and lipases in non-activated and activated HSCs revealed that, at the mRNA level, ATGL and LPL are highly expressed while expression levels of the remainder ester hydrolases (ES-4, ES-10, ES-3, AY034877 (Ces2A), D50580 (Ces2E), AB010635 (Ces2C), hepatic lipase, HSL, cholesterol ester lipase, and pancreatic triglyceride lipase) are very low or undetectable [89]. Interestingly, upon HSC activation, mRNA expression of ATGL decreases while that of LPL increases manifold [89]. Since activation of HSCs leads to RE mobilization [111,112] it suggests that ATGL may not be involved in this process. LPL is known to exhibit RE hydrolase activity [61] and to be expressed in HSCs [113]. Since LPL is a secretory protein [114], known to degrade lipoproteins, it appears more feasible that LPL is secreted from HSCs and is not active on cytosolic LDs and thus not responsible for RE mobilization. This aspect of intracellular LPL function, however, has thus far not been addressed.

A novel candidate for RE hydrolysis in HSCs was recently characterized by Pirazzi et al. [115]: PNPLA3 (also known as adiponutrin), the closest homologue of ATGL (annotated as PNPLA2), was initially described to exert triglyceride hydrolase [116,117] and lysophosphatidic acid acyltransferase activity [118]. In an association study, a mutation in the PNPLA3 gene, resulting in the amino acid change I148M, was found to correlate with liver fibrosis [119]. The study by Pirazzi et al. [115] showed that PNPLA3 is expressed in human primary HSCs and that its expression is upregulated by retinol and insulin, resulting in reduced LD content. Furthermore, PNPLA3 promotes the release of retinol from human HSCs and purified wild-type—but not mutant I148M PNPLA—hydrolyzes retinyl palmitate in a time- and dose-dependent manner [115]. Interestingly, cell lysates of hepatocytes overexpressing wild-type or mutant I148M PNPLA3 did not exhibit altered RE hydrolase activities [115]. Similarly, also COS7 cell lysates containing murine PNPLA3 did not show increased RE hydrolase activity [95] suggesting that PNPLA3 may not act as RE hydrolase in other cell types than HSCs. In a follow-up study, Pingitore et al. [116] showed that PNPLA3 expression in primary human HSCs is induced by the fibrogenic cytokine transforming growth factor-beta. Furthermore, expression of wild-type PNPLA3 but not mutant I148M reduces cellular neutral lipid content of the human hepatic stellate cell-line LX-2, and induces a reduction in the secretion of matrix metallopeptidase 2 as well as tissue inhibitor of metalloproteinase 1 and 2 indicative for an anti-fibrogenic role of PNPLA3 in HSCs [116]. Together, data of these studies [115,116] demonstrate that human PNPLA3 affects cellular LD content and retinol release of HSCs and that wild-type but not mutant I148M PNPLA3 exerts an anti-fibrogenic effect. The involvement of PNPLA3 in human retinoid metabolism was corroborated by the observation that carriers of a homozygous I148M allele exhibit lowered circulating RBP4 levels [115]. Although PNPLA3-deficient mice have been generated [120,121] they have not been reported to exhibit disturbed vitamin A homeostasis (e.g., altered circulating retinol or hepatic retinoid contents) which may be indicative of a minor role of PNPLA3 in murine liver retinoid homeostasis or even for a different role of PNPLA3 in mice and humans.

For the mobilization of REs of HSCs, an alternative process, in addition to the hydrolysis of cytosolic LDs by neutral lipid hydrolases, has been postulated. In 2011, Thoen et al. [122] first proposed a role for autophagy during HSC activation. The authors showed in an elegant experiment that pharmacological inhibition of autophagy by bafilomycin A1 resulted in decreased expression of HSC activation marker proteins [122]. Furthermore, after treatment of murine primary HSCs with platelet-derived growth factor-BB (PDGF-BB) the microtubule-associated protein light chain 3 beta (MAP1LC3B/LC3B), a regulatory protein for autophagosome formation and lysosomal fusion, co-localized with LDs [122]. This is interesting, since PDGF-BB was shown to act as a mitogen for HSCs and that infection of rats with an antisense PDGF-BB adenovirus resulted in decreased hepatic collagen deposition and alpha-smooth muscle actin (α-SMA) expression [123]. In summary, Thoen et al. [122] concluded that autophagic flux is increased during HSC activation. Hernández-Gea et al. [124] further investigated the role of autophagy in HSCs. They showed that administration of mice with the fibrosis-inducing agents (CCl_4_, thioacetamide) resulted in increased expression of autophagy markers (LC3, LC3II) in isolated primary HSCs. More interestingly, CCl_4_ treatment of mice lacking the autophagy mediator ATG7 specifically in HSCs (ATG7-(F/F)-glial fibrillary acidic protein (GFAP)-Cre mice) resulted in reduced expression levels of the fibrosis marker protein α-SMA and the matrix protein collagen 1 in primary HSCs. Furthermore, incubation of HSCs with the autophagy inhibitor 3-methyladenine or silencing of *Atg7* led to increased LD area, suggesting that LDs of HSCs are—at least in part—degraded through the autophagosomal machinery. The authors [124] further concluded that selective reduction of autophagy in HSCs might be a treatment strategy for fibrotic liver disease. Although GFAP has more recently been shown not to be expressed in HSCs [125], questioning the applicability of the GFAP-Cre mouse model for an HSC-specific knock-out, the involvement of autophagy in the degradation of cytosolic LDs (lipophagy) is increasingly growing [105,106,126,127].

### 3.3. RE Hydrolases of Other Non-Parenchymal Cells

Rat primary Kupffer- and sinusoidal endothelial cells together with parenchymal cells are known to accumulate considerably less REs than HSCs (~10-fold less) [17,57]. For the hydrolysis of REs, Kupffer- and sinusoidal endothelial cells comprise intrinsic hydrolytic activity which in rat primary Kupffer- and endothelial cells was found to be ~5–10 less than that of hepatocytes and ~8–40-fold less than that of HSCs [57,128]. Studies on the identity of RE hydrolases in these cell types are rare. At the mRNA level, Kupffer cells have been found to express, at high levels, ATGL and LPL. At a very low level, ES-4 is expressed [89]. Similarly, endothelial cells were found to express ATGL at a higher level and Es-4, and Es-10 at lower levels [89]. Further details on RE hydrolases in these cell types have not been explored to date.

**Figure 2:**
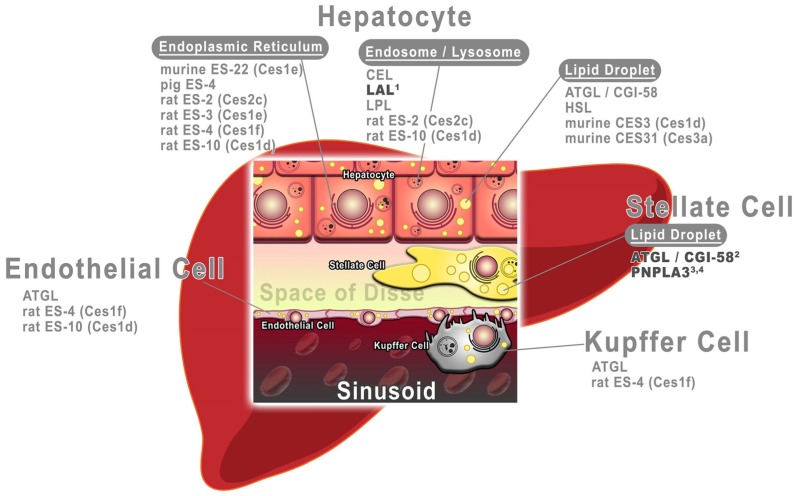
Depiction of hepatic enzymes of different liver cell types and organelles, known to exhibit retinyl ester hydrolase activity. ATGL, adipose triglyceride lipase; CEL, carboxyl ester hydrolase/lipase; CES3, 31, carboxylesterase 3, 31; CGI-58, comparative gene identification-58; ES-1, 2, 3, 4, 10, 22, esterase 1, 2, 3, 4, 10, 22; HSL, hormone-sensitive lipase; LAL, lysosomal acid lipase; LPL, lipoprotein lipase; PNPLA3, patatin-like phospholipase domain containing 3. Enzymes which have been demonstrated to affect cellular retinoid homeostasis of respective liver cell type are indicated in bold. Footnotes: 1. Grumet et al. J Biol Chem. 2016 19:17977-87; 2. Taschler et al. Biochim Biophys Acta 2015 1851:937-45; 3. Pirazzi et al. Hum Mol Genet. 2014 23:4077-85; 4. Pingitore et al. Hum Mol Genet 2016 ahead of print.

In summary, a large number of enzymes has been identified to be expressed in various liver cell types and to exhibit RE hydrolase activity (see Figure 2). Interestingly, a functional role in liver cell RE homeostasis has been demonstrated only for a few of these enzymes, including the lysosomal protein LAL of hepatocytes and two patatin-like phospholipase domain-containing proteins, ATGL and PNPLA3, of stellate cells (indicated in bold in Figure 2). To date, however, no rate-limiting role in the hydrolysis of hepatic REs has been established for any of these enzymes in an animal model. Thus, the rate-limiting enzymes in liver RE mobilization still need to be discovered.

## 4. Pathophysiological Processes Associated with Mobilization of Hepatic RE Stores

Hepatic RE stores are known to outbalance fluctuations in nutritional vitamin A intake. Under times of nutritional vitamin A undersupply, hepatic RE stores are mobilized to maintain constant circulating retinol levels. For example, rats fed a vitamin A-deficient diet maintain constant circulating retinol levels over a period of 84 days [9]. Upon depletion of hepatic RE stores after 97 days of a vitamin A-deficient diet (3.4% are left) also a drop in circulating retinol levels was observed. As is also evident from this study [9], the majority of hepatic REs are mobilized from the non-parenchymal cell fraction in which RE content decreased by ~98%. In addition to the mobilization of hepatic RE stores as a consequence of vitamin A undersupply, hepatic RE stores have also been found to be depleted under pathological conditions in response to certain forms of liver damage [10,11,12,129]. Below we will explore several forms of liver injuries that have been shown to be associated with a loss of hepatic vitamin A content. Since liver injuries derive from various sources and effectors, these sections only cover some of the possible scenarios.

### 4.1. RE Mobilization upon Alcohol-Related Liver Diseases/Alcoholic Liver Cirrhosis

In 2010, alcoholic liver cirrhosis caused almost 500,000 deaths worldwide [130]. Alcoholic liver cirrhosis accounts for around 48% of all liver cirrhosis deaths [130]. In a study conducted in 1979, McClain et al. [131] found that alcoholics suffer from vitamin A deficiency-like symptoms, such as night blindness or hypogonadism. Two years later, two landmark studies conducted by Lieber and colleagues demonstrated the long assumed correlation between alcohol consumption and depletion of hepatic vitamin A stores: In the first animal study, Sato and Lieber [132] reported hepatic vitamin A depletion after chronical ethanol consumption in baboons and rats. The follow-up study in 1982 investigated hepatic vitamin A levels of (human) alcoholics and found them to be significantly decreased in people with alcohol-attributable liver damage [133]. Among 59 patients tested, the hepatic vitamin A levels of patients diagnosed with alcoholic fatty liver were significantly lower, even when compared to subjects suffering chronic persistent hepatitis [134]. In patients with alcoholic hepatitis, hepatic vitamin A content was decreased even further to 1/10th of that of “normal” livers (~500 µg vitamin A/g wet liver weight) [134]. Patients who had developed alcoholic cirrhosis exhibited the lowest vitamin A levels which were only a few percent compared to the values of the control group [134]. Another study in 1989 reported hepatic retinol and RE levels of patients with alcoholic liver disease and found that the total hepatic retinoid levels were lower in patients with alcoholic cirrhosis (15 subjects) as compared to patients suffering other liver diseases such as nonalcoholic fatty liver or chronic cholestasis [135]. Follow-up studies revealed that alcohol intake in fact decreased vitamin A levels of HSCs in men [136]. These circumstances hint at an association between alcohol-attributable liver damage and the activation of HSCs. One study [129] found a correlation between the number of HSCs and the progression of alcoholic liver disease: Depending on the severity of disease progression, patients showed a significant decline in the amount of HSCs per mm^2^ liver section [129]. The authors speculated that this phenomenon could be due to the activation of HSCs and their trans-differentiation into myofibrobast-like cells. While this seems plausible, definite data to support this association is lacking.

An induction of HSC neutral RE hydrolases (expression or activity) during the progression of alcohol-induced liver damage seems plausible, since studies in animals and humans have shown a similar decline in hepatic retinoid levels after chronic alcohol consumption and progression to liver fibrosis [129,132,134,135]. These observations are consistent with the activation of HSCs. The hypothesis that ethanol leads to the mobilization of hepatic REs by neutral RE hydrolases is further bolstered by the observation of Friedman et al. [137]. The authors demonstrated in an in vitro experiment that addition of ethanol to rat liver homogenates stimulated neutral RE hydrolase activity [137]. Stimulation became significant at a concentration of 0.1 M ethanol in rat liver homogenates which equals the concentration in the circulation of rats after acute ingestion of alcohol (6 g/kg bodyweight, [138]).

### 4.2. RE Mobilization upon Nonalcoholic Fatty Liver Disease

NAFLD is the most common chronic liver disease in the Western world, with a prevalence of approximately 30% in adults and 10% in children and adolescents [139]. NAFLD essentially describes a state of excessive fat deposition in the liver with no history of excessive alcohol consumption. According to a practice guideline published in the *Journal of Hepatology*, diagnosis of NAFLD requires that (i) there is evidence of hepatic steatosis either by imaging or by histology; and (ii) there are no causes for secondary hepatic fat accumulation such as significant alcohol consumption, use of steatogenic medication or hereditary disorders [140]. Furthermore, NAFLD is strongly associated with features of the metabolic syndrome and can be viewed as the hepatic manifestation of insulin resistance [141,142]. Similar to alcohol-induced liver damage, long-term consequences include hepatitis (referred to as non-alcoholic steatohepatitis, NASH) which further progresses to liver fibrosis and ultimately liver cirrhosis. As already outlined above, these conditions involve the activation of HSCs. Thus, it seems obvious that depending on the degree of NAFLD, decreased hepatic vitamin A levels are to be expected. In fact, a recent study addressed this very matter by investigating liver vitamin A reserves in 68 patients with NAFLD and class III obesity [143]. Authors found that 67.6% of the patients exhibited inadequate liver vitamin A reserves (≤20 μg/g tissue), while 26.5% of the patients were found to have liver reserves of vitamin A classified as critical (0.6–5 μg/g tissue). Furthermore, the liver reserves of 19.1% of patients were close to being absent (<0.6 μg/g tissue) [143]. In addition, the same study demonstrated an association between liver vitamin A levels and the severity of NAFLD. Specific RE levels were not reported in the study. It is important to point out that the authors did not conclude from their findings that the drop in hepatic vitamin A levels was due to activation of HSCs. Instead, authors hypothesized that decreased hepatic vitamin A content was due to increased oxidative stress in livers of patients with NAFLD. Vitamin A has been shown to act as an antioxidant [144], which is why authors suggested a coherent consumption of hepatic vitamin A by reactive oxygen species (ROS) [145]. In return, hepatic ROS has been previously reported to contribute to HSC activation [146] involving ROS-sensitive cytokines which are released from immune cells during inflammation [147]. If the liver has a higher demand for the antioxidant retinol, the mobilization of RE reserves stored in lipid droplets of HSCs would be a likely scenario. Although in some NAFLD subjects [143] decreased hepatic RE content may be a result of HSC activation, other NAFLD subjects have actually been reported to exhibit the opposite, increased hepatic RE content [148]. The inherited rs738409 C→G polymorphism which encodes for the isoleucine-to-methionine substitution at residue 148 (I148M) of PNPLA3, is known to be associated with increased liver fat content and the development of NAFLD (see also Section 3.2 above) [119]. Although PNPLA3 has been shown to exhibit acyltransferase [118] as well as neutral lipid hydrolase activity [115,116,117,149], the underlying molecular mechanism for the strong NAFLD predisposition is presently unclear. Hepatic neutral lipid accumulation in subjects homozygous for the rs738409 C→G polymorphism could be explained either by a gain of acyltransferase activity [118] as well as a loss of neutral lipid (triglyceride and/or RE) hydrolase activity [115,116]. Despite the lack of an exact molecular mechanism, homozygous subjects for the I148M mutant allele exhibit lower fasting circulating retinol levels (while RBP4 levels were unchanged) and elevated hepatic RE content [148,150] suggesting that disturbed hepatic vitamin A mobilization may be one of the underlying causes.

### 4.3. RE Mobilization upon Viral Hepatitis

Kataria et al. [151] investigated hepatic vitamin A stores during progression of liver disease in patients with chronic hepatitis C virus infections (HCV). The authors investigated the relationship between retinoid/carotenoid concentrations in serum and hepatic tissue and examined the relationship between retinoids and HSC activation in patients suffering HCV [151]. While the study revealed a weak negative trend of hepatic retinyl palmitate content with increasing fibrosis stage, retinyl palmitate levels were inversely and significantly correlated with hepatic α-SMA expression, a reliable marker for HSC activation [151]. Santana et al. [152] investigated free hepatic retinol levels in non-cirrhotic patients infected by HCV and found a negative trend in patients with more advanced HCV-induced liver damage. Another study [153] investigated liver vitamin A reserves using the relative dose response method, a non-invasive test that allows indirect estimation of total hepatic vitamin A content by comparing plasma retinol levels before and after acute retinyl palmitate ingestion. In a group of 144 patients infected with HCV, 34% who participated at the test showed inadequate liver vitamin A reserves [153].

In summary, HSCs represent the most important player in the progression of liver fibrosis. Advanced stages of fibrosis reportedly lead to the loss of hepatic vitamin A content which is mostly stored in cytosolic LDs of HSCs. This mobilization is presumably due to an induction of RE hydrolase(s) (activity and/or expression), while the rate-limiting enzymes responsible are currently unknown.

## 5. Conclusions

Over the last few decades, the generation of transgenic and knock-out mouse models as well as the development of techniques for the overexpression/silencing of genes in cultured cells has significantly advanced the understanding of protein function. Some attempts have been made for the understanding of the physiological role of established lipases in retinyl ester (RE) metabolism. Despite the progress in understanding the limiting role of, e.g., hormone-sensitive lipase in adipose tissue RE mobilization, the limiting role of any of the hepatic hydrolases/esterases/lipases in liver or a specific liver cell type is missing. Profound knowledge about the mechanisms and enzymes involved in the mobilization of hepatic RE stores might likely expand beyond satisfying curiosity and has the potential to unravel new targets for treating pathophysiological conditions under which hepatic vitamin A is adversely mobilized. Particularly, RE hydrolases of hepatic stellate cells (HSCs) have a fair potential to be useful targets for pharmaceutical interventions. Assuming that such neutral RE hydrolase(s) of HSCs do exist, future investigators will have to think of experimental approaches that allow identification of hitherto unknown or uncharacterized proteins.
